# Pronounced Enhancement in Radiosensitization of Esophagus Cancer Cultivated in Docosahexaenoic Acid via the *PPAR -*γ Activation

**DOI:** 10.3389/fmed.2022.922228

**Published:** 2022-07-22

**Authors:** Ying Yang, Ying Xu, Congzhao Zhao, Lirong Zhang, Aslibek Nuerbol, Lili Wang, Yang Jiao

**Affiliations:** ^1^State Key Laboratory of Radiation Medicine and Protection, School of Radiation Medicine and Protection, Soochow University, Suzhou, China; ^2^Department of Ultrasound Diagnosis, Gaochun Peoples' Hospital, Affiliated Hospital of Nanjing Drum Tower Hospital, Nanjing, China; ^3^Department of Radiotherapy, Second Hospital of Soochow University, Suzhou, China

**Keywords:** esophageal cancer (EC), docosahexaenoic acid-DHA, prognosis (carcinoma), radiosensitivity, DNA damage

## Abstract

Docosahexaenoic acid (DHA) has been reported to suppress the tumor growth and improve prognosis and has been used to cooperate with many other chemotherapy medicines. Up to now, surveys focused on the Interaction between DHA and radiation are relatively modest. Our study sought to evaluate the radiosensitivity changes caused by DHA on esophageal cancer cells. We selected TE-1 and TE-10 esophagus cancer cells as models and performed routine cell proliferation assay and cloning assay to detect the impact of DHA combined with X-ray. We used cell cycle assay, lipid peroxidation assay, comet assay, and apoptosis assay to unearth the potential causes. We also launched a mouse transplanted tumor experiment to verify the synergetic effect of DHA and irradiation. Finally, a western blot assay was used to find a novel mechanism. As a result, DHA improved TE-1 and TE-10 radiosensitivity *in vivo* and *in vitro*. What's more, *PPAR-*γ expression increased due to the DHA supplement. Inhibiting *PPAR-*γ could attenuate benefits brought out by DHA somehow. Due to its explicit usage and convenience, DHA would serve as an adjuvant therapy before radiotherapy if the clinical trials indicated positive.

## Introduction

Esophageal cancer has become the 7^th^ most common malignant solid tumor worldwide, but it has a poor prognosis that ranks 6^th^ from the bottom (1, 2). Its typical histopathologic types can be classified into esophageal squamous cell carcinoma (ESCC) and esophageal adenocarcinoma (EA). The former occupied the absolute majority before the 1970s. Since then, however, the incidence of esophageal adenocarcinoma has risen year by year (2, 3). Especially in the UK and some other Western Europe countries, the morbidity of EAC has caught up with ESCC (3). These risk factors, which are the main ones responsible for ESCC, are listed as Alcohol and Smoking. In the meantime, Alcohol and smoking are also relative to the onsets of EA (4, 5). In addition, Barrett's esophagus and obesity are the other two leading causes of EA (6). Most ESCC's predilection sites are in the middle and upper esophagus, but the EA primarily occurs in the gastro junction. These two kinds of esophageal carcinomas share clinical signs, such as dysphagia, weight loss, and thoracalgia.

Due to the insidious onset of esophagus tumor, the diagnosis is often delayed to the middle and late periods so that the optimal opportunity for treatment would be delayed. Surgery is the traditional choice for therapy, for it can put more concentration on the solid tumor than other means. However, the 5-year overall survival is poor since the tumor cells would locally recur and distant metastasize after the primary surgical resection of cancer lesions (5, 7). Historically, radiotherapy (RT) has been exploited as a valid treatment, particularly for those who are not applicable for surgical operations. Lately, some studies have demonstrated that partial RT could control the primary tumor combined with some other symptoms to prolong survival time somehow (8, 9). However, it bothers clinicians that radiotherapy may produce some side effects and its efficacy in advanced esophagus tumors remains to be improved (10). Thus, RT could be an effective method to be taken advantage of together with other auxiliary measures.

Docosahexaenoic acid (DHA) is a long-chain n-3 polyunsaturated acid (n-3 PUFAs) and enriched in marine food products such as kelp and abyssal fishes, together with the linoleic acid family (n-6 PUFAs) constitute the unsaturated fatty acids. It has been confirmed that n-3 PUFAs have anticancer activity in many systems (11–13). n-3 PUFAs can partly prevent tumorigenesis (14), inhibit cancer cell proliferation and refrain tumor growth (7, 15). After almost a decade of prospective cohort investigations conducted by the USA National Cancer Institute, they found that marine food intake was associated with a 20–27% lower risk of EA and head-neck tumors (HNC), but not ESCC (16).

This study investigates whether DHA could cooperate with radiation, one of the main therapeutic methods, to improve the esophagus squamous cell's death or inhibit its proliferation. Our results indicated that DHA further promoted radiation-induced cell viability inhibition and cell death by prolonging G2/M arrest and apoptosis *in vivo* and *in vitro*. In conclusion, our study provides a piece of clinical evidence manifesting that radiotherapy and DHA used in conjunction could be a novel remedy for ECCs treatment. Furthermore, *PPAR-*γ (peroxisome proliferators-activated receptors), a ligand-activated transcription factor that regulates lipid metabolism (17), was proposed that could take participant in this mechanism, and its suppression would inhibit the radiosensitization of DHA in turn.

## Results

### DHA Reduces Cell Viability After Irradiation in Esophagus Cancer Cells

**Figure 1:**
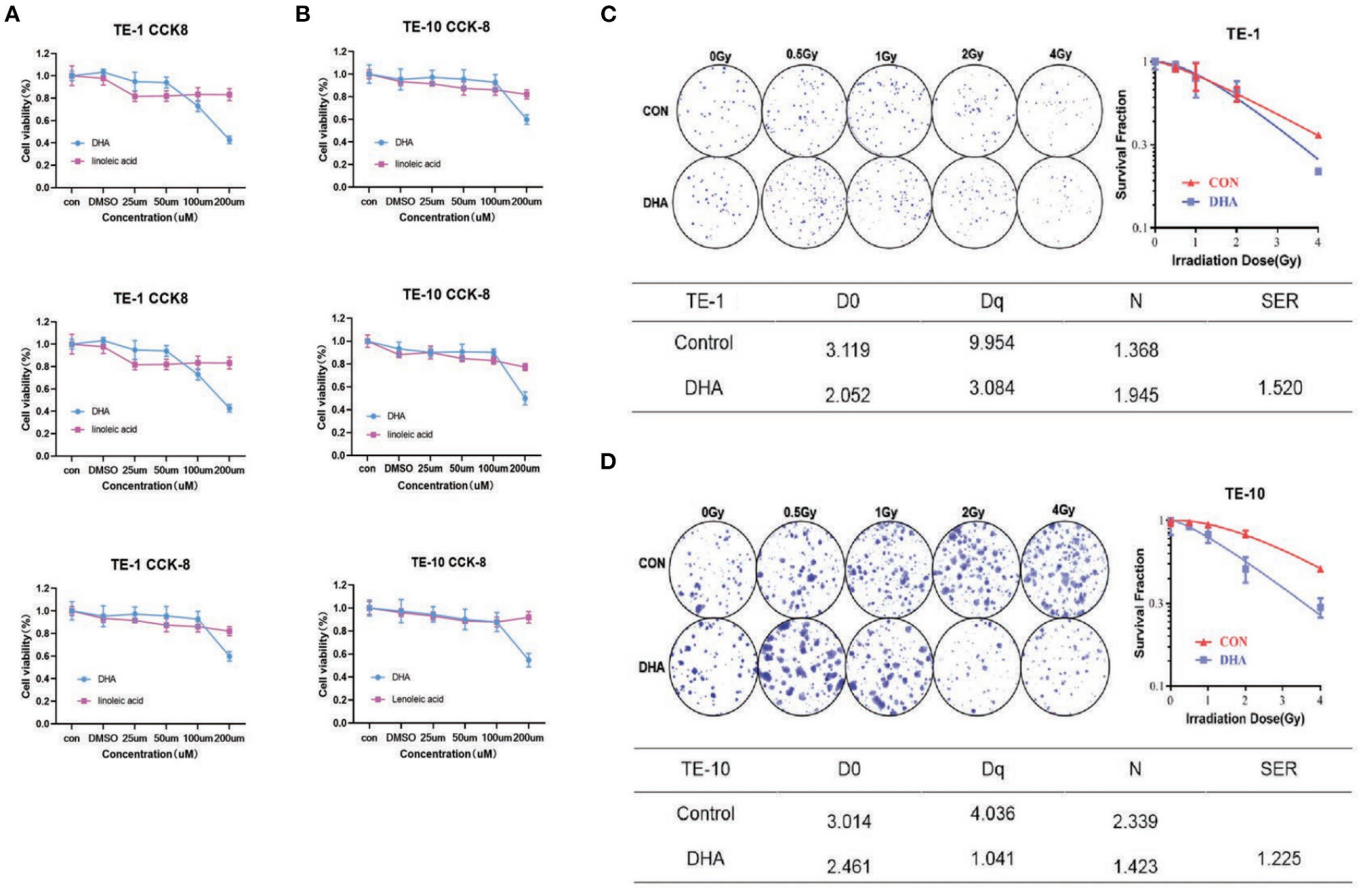
DHA reduced cell viability after irradiation in esophagus cancer cells. TE-1 and TE-10 cell viability was detected 24h after co-incubated with 0, 25, 50, 100, and 200uM DHA and LA. Panels showed three independent experimental results of cell viability detected by CCK-8 kit **(A,B)**. Representative clones in every group after 0, 0.5, 1, 2, and 4Gy radiation were listed in this figure. The corresponding clonogenic cell survival curves were pictured after the cloning formation counts normalized to the unirradiated cells **(C,D)**. We used the multi-target, single-hit model to measure the sensitizer enhancement ratio (SER).

First, to determine the concentration of PUFAs we used, the CCK-8 assay was performed to investigate the toxicity of PUFAs upon esophagus cells. TE-1 and TE-10 cells were cultured with different concentrations (0–200μM) of DHA and linoleic acid(LA) for 24h. According to the results, DHA and LA inhibited the esophagus cell viability with a dose-dependent relationship (Figures 1A,B). When the TE-1 and TE-10 cells inhibiting rate reached 20%, the concentrations of DHA were 100 and 150 μM, and the same concentrations of LA were picked as the matched group.

The colony formation assay was carried out with the indicated DHA and LA concentration to verify whether DHA has a combination effect with IR. With the dose of radiation given increased (0–4Gy) after incubation with DHA and LA, the ability to form cells colony decreased. The sensitizer enhancement ratio (SER) was 1.520 (*P* < 0.01) in TE-1 cells and 1.225 (*P* < 0.01) in TE-10 cells, implicating possible reciprocity between DHA and irradiation (Figures 1C,D).

### DHA and IR Combination Enhances DNA Damage in Esophagus Cancer Cells

**Figure 2:**
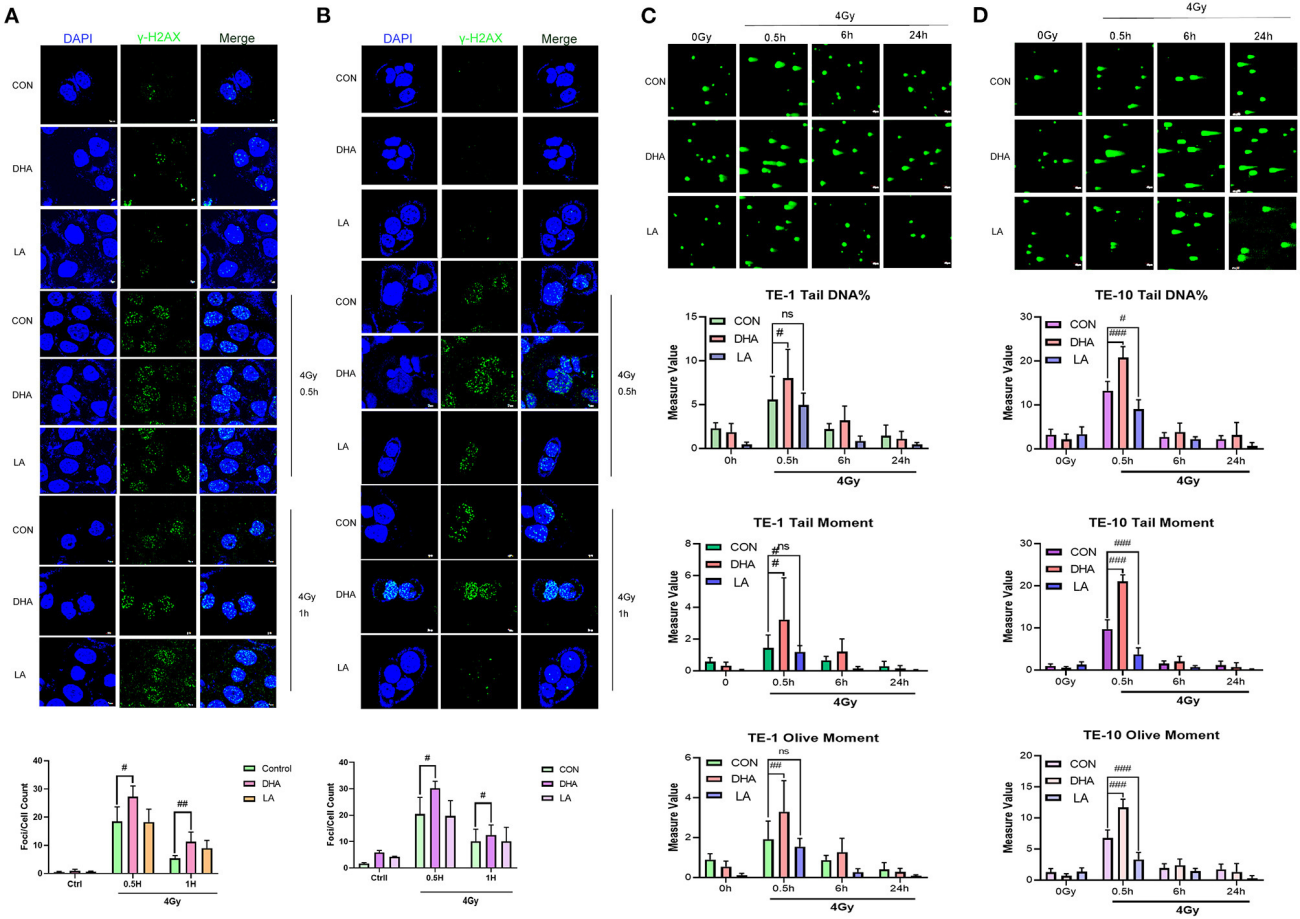
DHA and IR combination enhanced DNA damage in esophagus cancer cells. Immunofluorescences photos of γ-H_2_AX in TE-1 and TE-10 cells after 4Gy irradiation at 0.5h and 1h. The scale bar represents 7μm **(A,B)**. The comet assay was carried out to measure the DNA damage of IR. Cells were pretreated with DHA and LA for 24h and then exposed to 4Gy irradiation. Cells were gathered at different points of time after irradiation. Scale bar represents 40μm **(C,D)**.

The obstruction of DNA repair and aggravated DNA damage could decline cell proliferation. We used the immunofluorescence staining assay to detect γ-H_2_AX quantity, which was one of the most commonly used markers of DNA damage, as well as the Single cell gel electrophoresis (SCGE). TE-1 and TE-10 cells were well treated with DHA and LA according to the concentration mentioned above for 24h. After 4Gy irradiation, the cells were immobilized and stained at different time points. All the groups generated the most extensive DNA fragmentation index at 0.5h after the irradiation (Figures 2A,B). The DHA and X-ray combination reached a higher fragment index than the others in these two esophagus cancer cells at 0.5h post-irradiation (*P* = 0.044 in TE-1, *P* = 0.035 in TE-10). In the meantime, there was no statistical difference between the control and LA+IR group (*P* = 0.6119). This observation was also confirmed in a comet cell assay. DHA and radiation combination group owned the highest Tail DNA%, Tail moment, and Olive moment (Figures 2C,D). The DNA damage peaked at 0.5h after radiation can be concluded from these figures. Compared to the control group, DHA and X-ray combination sharply increased the Tail DNA%, Tail moment, and Olive moment at 0.5h post-irradiation (*P* < 0.005). Of note, there were also statistical differences between the control and LA+IR group (*P* < 0.005). However, these differences appeared to be a decline in the damage to DNA.

### DHA Increases G2/M-Phase Arrest and Apoptosis Caused by IR

**Figure 3:**
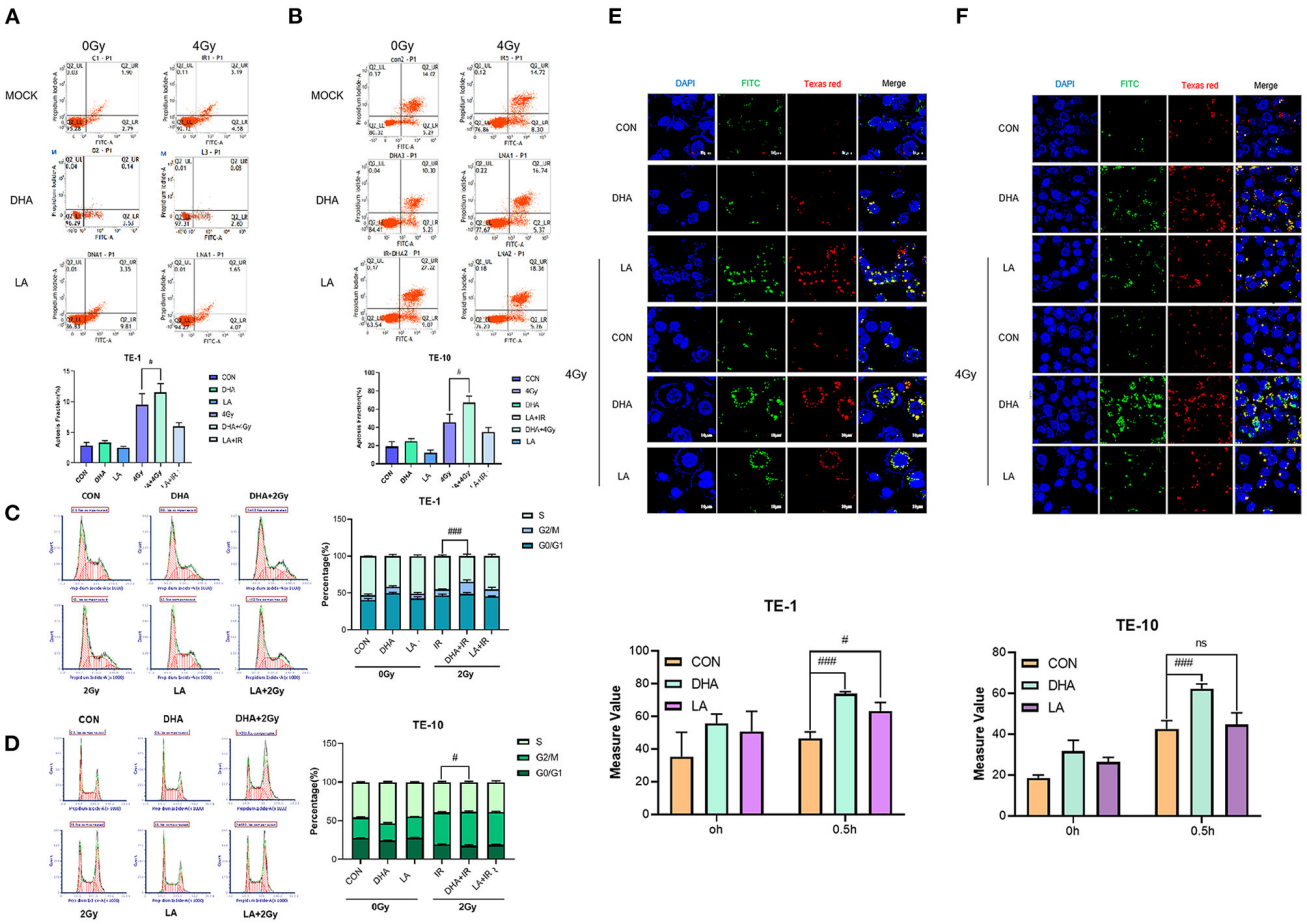
DHA improved apoptosis, G2/M arrest and lipid peroxidation caused by IR. Cell apoptosis was measured by Annexin V/7-AAD double staining kit according to the instruction. The data were exhibited as the mean ± s. e. m. of three independent experiments **(A,B)**. Cell cycles were detected by flow cytometry 24h after 2Gy irradiation. Both attached and flowing cells were collected, #*P* < 0.01 compared with the control group of cells **(C,D)**. Typical photomicrographs of BODIPY fluorophore 581/591 staining of lipid droplets in cells at 0.5h after 4Gy irradiation. Cells were pretreated according to the methods recorded before **(E,F)**.

We used flow cytometry to measure the cell apoptosis at 24h after 4Gy irradiation. DHA and radiation combination brought a higher cell apoptosis rate in TE-1 and TE-10 than in the only DHA group or radiation group (Figures 3A,B), and the *P* values were 0.0338 and 0.0161 separately. Same as before, this circumstance does not repeat in LA and the radiation combination group. Moreover, the apoptosis population shows a downward trend, although there was no significance between LA+IR and the control group (*P* = 0.1862 in TE-1, 0.1962 in TE-10).

To investigate whether server proliferation suppression caused by DHA had something to do with cell cycle progression, we implemented 2Gy X-rays on TE-1 and TE-10 cells after pretreated with DHA and LA for 24h. Radiation alone had a G2/M arrest effect upon esophagus cancer cells (Figures 3C,D), while the combination of radiation and DHA brought a more extended block (*P* < 0.005). The same did not happen in the LA and radiation united treatment group, and the *P* values were 0.6350 and 0.5342.

We took advantage of BODIPY 510/590 dyestuff to explore lipid peroxidation after the irradiation. The result showed that radiation rapidly enhanced lipid peroxidation in TE-1 and TE-10 cells (Figures 3E,F). Besides this, both DHA and LA addition intensified the lipid peroxidation, and the former combination was higher than the latter. The *P* values of the DHA+IR group were 0.0003 and 0.0008, while the LA+IR group were 0.0261 and 0.8286.

### DHA and Radiation Combination Augments the Expression of PPAR-γ

**Figure 4:**
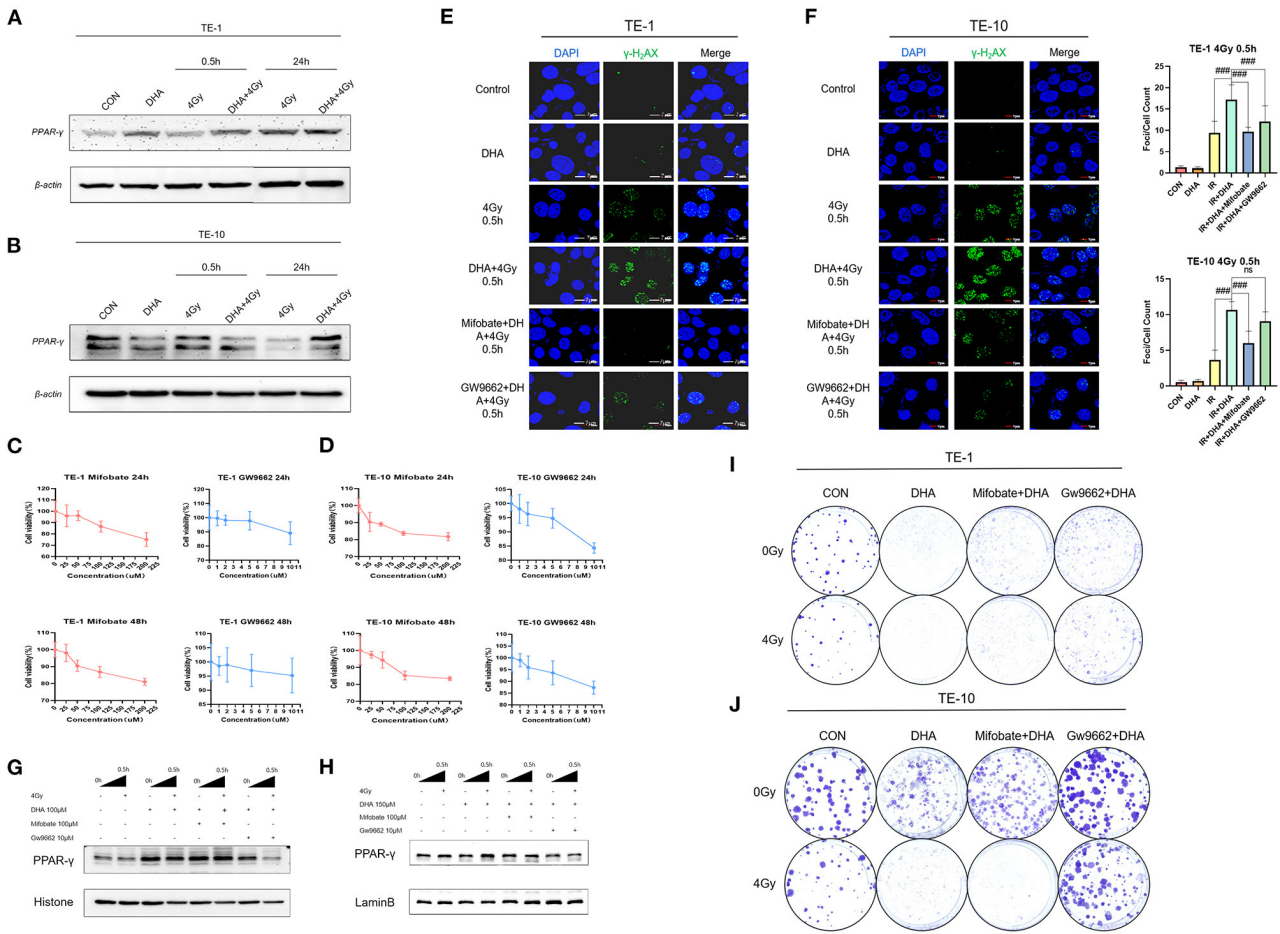
DHA and IR combination augmented the expression of *PPAR-*γ, inhibiting *PPAR-*γ could partly reversed the sensitization. Western blot was performed to determine the expression of *PPAR-*γ of TE-1 and TE-10 cells at different points of time after irradiation **(A,B)**. The esophagus cancer was co-incubated with Mifobate and GW9662, cells viability was calculated by the CCK-8 assay at 24h and 48h later **(C,D)**. DNA damage was detected by γ-H_2_AX immunofluorescence 0.5h after irradiation in TE-1 and TE-10 cells which got cocultivation with Mifobate and Gw9662 anterior to DHA and IR combination. Scale bar represents 7μm **(E,F)**. To verify the suppression of *PPAR-*γ induced by the inhibitors Mifobate and Gw9662, the western blot was used to show the nuclear *PPAR-*γ *expression* **(G,H)**. Representative clones of TE-1 and TE-10 exposed to 0 or 4 Gy after the cocultivation of DHA and the *PPAR-*γ inhibitors **(I,J)**.

*PPAR-*γ is one of the nuclear ligands that fatty acids can activate. To investigate whether the improvement of radiosensitivity in TE-1 and TE-10 induced by DHA has some relationships with *PPAR-*γ, we used a western blot assay to detect the protein expression. The result shows that DHA alone did not affect the expression apparently, but DHA and IR combined did. The *PPAR-*γ expression was enhanced after irradiation, indicating activation of *PPAR-*γ (Figures 4A,B).

### PPAR-γ Inhibition Attenuates DHA -Mediated Radiosensitization

To explore whether the radiosensitizing effect of DHA owes to *PPAR-*γ, we incubated TE-1 and TE-10 cells with Mifobate and GW9662, two kinds of *PPAR-*γ antagonists, for 24h before being applied with DHA and radiation. Firstly, we used the CCK-8 assay to establish the working concentration of Mifobate and GW9662 (Figures 4C,D). Then immunoblotting was carried out to guarantee that *PPAR-*γ was suppressed, which was in line with our anticipation. The expression of *PPAR-*γ accumulated in the cell nucleus was markedly cut down (Figures 4G,H). The immunofluorescence assay was then repeated to observe the γ-H2AX, a marked indicator of DNA damage, at 0.5 h after 4Gy irradiation (Figures 4E,F). The result showed that the focis per cell increased and peaked at 0.5h after the irradiation, which was consistent with our previous outcomes (*P* < 0.005). Besides this, exposure to DHA sharply increased the foci counts post-irradiation(*P* < 0.005).

Moreover, Mifobate and Gw9662 twisted the tendency, and Gw9662 played a more vital role. The *P* values of Mifobate supplementary all <0.005 compared to the DHA+IR group. Cell clones were generated after treatment of Mifobate and Gw9662 for 24h before the Interaction of DHA and radiation. The clone formation fraction sharply decreased in the DHA and IR combination group, the same as before. The effect of cell-killing ability was reversed in Mifobate and Gw9662 application groups. The Gw9662 showed a more obvious resistance to radiotherapy that contributes to the proliferation of esophagus cancer cells (Figures 4I,J). These results indicate that inhibition of *PPAR-*γ could relieve the DNA damage caused by DHA and IR co-treatment and somehow relieve the radiosensitization induced by DHA.

### DHA and Radiation Combination Promotes Cell Death *in vivo*

**Figure 5:**
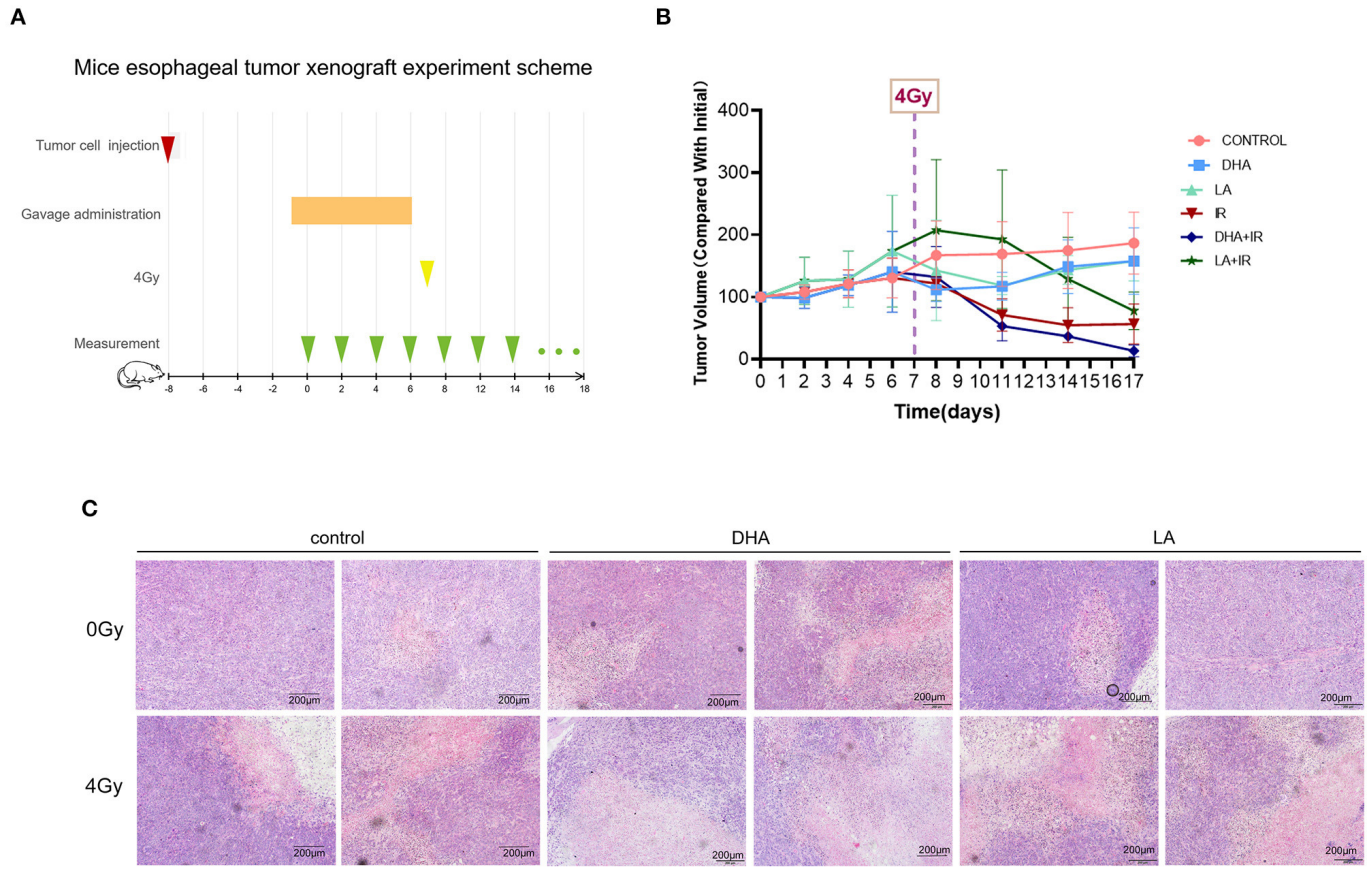
The scheme and results of esophagus tumor subcutaneous xenograft experiment. Every group consisted of 5 mice. TE-1 cells were inoculated under the skin of the nude mouse. DHA and LA were given to the mouse by gavage once a day for 1 week before radiotherapy. After irradiation, tumor volumes were recorded every 2 days **(A)**. The tumor volume was measured daily after TE-1 cells were injected and at 2-day intervals treated with 4 Gy irradiation **(B)**. HE staining was used to observe the pathological characteristics of transplantation tumors. Scale bar represents 200μm **(C)**.

Five-week-old female nude mice were injected subcutaneously with TE-1 cells [1 × 10^6^ in 100 μL] and gavaged with ddH_2_O, DHA, and LA (dosage regimen described later). After the treatment course (Figure 5A), the tumors were exposed to a 4Gy X-ray-targeted radiotherapy. The DHA and radiation combination slowed down the tumor growth relative to the other groups (Figure 5B). However, there was no statistical difference between the IR only and the DHA+IR group (*P* = 0.2065~0.9589). We performed HE staining to compare the malignant degree among the control, DHA, and LA treatment groups. Consistent with the *in vitro* results, the DHA and radiation combination shows the lowest malignancy compared with other groups. A few cancer cells can be found in the view of a microscope (Figure 5C).

## Discussion

Radiotherapy is a conventional clinical treatment for esophagus cancer. However, this treatment can cause side effects such as esophagitis and digestive symptoms (18). Besides, because the esophagus cancer cells are not very sensitive to radiation, the curative effect of radiotherapy remains enhanced. Therefore, combination with other agents which could solve these problems was sought-after. N-3 PUFAs, especially DHA, have benefited the human body in many aspects (12). It is a dietary component, and the amount of guidance is noticeable. At the same time, DHA has been well-focused in recent years, owing to its antitumor potential (19). Moreover, several studies have proved that DHA can synergize with other drugs like Oxaliplatin, Adriamycin, and Cisplatin nowadays (15). However, there is still little research about the combination use of DHA and radiation. The current study investigated whether DHA has reciprocity with radiation upon ECGG treatment.

Our results showed that DHA with radiation combination decreased cell viability in the ECGG cell lines. Interestingly, the other PUFAs, LA alone or combined with radiation, did not trigger the same phenomenon. Considering that DHA could relieve inflammation and other side effects caused by chemotherapy in esophagogastric adenocarcinomas, it makes sense that DHA would enhance the efficacy of radiotherapy upon ECGG (20).

For now, there's only two types of sensitizers achieved clinical-approval, one is the halogenated pyrimidine compound, the other one is hypoxic cell radiation sensitizer. 5-iododeoxyuridine is one of the representatives of halogenated pyrimidine compounds, in an athymic nude mice human colon cancer xenograft model, the SER ranged from 1.20–1.48 in HCT116(MMR-) tumor xenografts and HCT116/3-6(MMR+) tumor xenografts while getting 2Gy or 4Gy irradiation. In another nude mice human glioma tumor xenograft model, the SER found with the combination of p. o. 5-iodo2-pyrimidinone-2'-deoxyribose (IPdR)+XRT was 1.3 (*P* = 0.05) (21). Misonidazole (MISO) belongs to the hypoxic cell irradiation sensitizers, which is known to be an outstanding sensitizer with SER = 1.8. Besides of the medicines we listed above, several novel sensitizers are being developed. Golden nanoparticals (GNPs) with acid-induced were reported to sensitize the combination therapy effect of RT and photothermal therapy (PTT), and the SER was calculated to be 1.52 (22).

DHA was a non-toxic nutrient, the human consumption has been granted GRAS (generally recognized as safe) status by the FDA (23). What's more, cell content of DHA can be increased in cultured cells by simply adding of DHA to the medium, so were animal tumor tissues (24). On basis of these studies, we consider that DHA was a decent radiation sensitizer in TE-1 and TE-10.

DNA breakage is the primary damage caused by radiation in cells, and DNA double-strand breaks (DSBs) are the most lethal damage form (25). Here, we used the γ-H_2_AX to mark the DSBs, which is the γ-phosphorylation product of H_2_AX after irradiated and will be located at the breakpoint. The population is positively associated with the number of DSBs. DHA increased the DSB induced by radiation while LA did not. Contemporaneously, the comet trailing length of the DHA and radiation combination group observed under the fluorescence microscope was the longest. Of note, both γ-H_2_AX staining and comet assay indicated that DSB took shape quickly after the exposure and was dismissed at 24h by and large, which means DSB gets repaired.

Once the destruction is repaired unsuccessfully, cells are subjected to begin programmed death or apoptosis (16). Our data illustrated that DHA and radiation combination induced more apoptotic cells via blocking the G2/M phase, which was consistent with our previous study that DHA could arouse cell apoptosis in breast cancer cells and the human B-cell lymphoma lines (26). What may be responsible for the enhancement in apoptosis is lipid peroxidation. As measured in this study, the DHA and radiation combination has sharply increased the level of lipid peroxidation compared with the control group.

The LA and radiation combination improved the lipid peroxidation level in the meantime, but not so efficient as DHA and radiation combination and didn't affect the cell viability according to our outcomings. At the same time, LA combined with irradiation appeared not statistically different from the only irradiation group in either the G2/M phase of cell apoptosis rate. These differences between DHA and LA may partly be caused by the DHA(n-3 PUFA) being more lipophilic than LA(n-6 PUFA), representing faster incorporation into the cell membrane and quicker oxidation (18). LA was reported to decrease the expressions of cyclin A, B_1_, and D_1_, increasing tumor cell accumulation in the G0/G1 phase, leading to human mammary adenocarcinoma cell proliferation suppression (27, 28). LA could also restrain the colorectal carcinoma cell growth, but no effect was observed on the cell cycle, similar to our experiment outcomings. Notably, all the inhibition of cell proliferation was most obviously at 48h after the co-incubation.

It is worth mentioning that compared with other n-3 PUFAs, DHA showed the most robust capability of tumor inhibition, which seems to be proportional to their double bond number. (29) Thus far, DHA was reported to co-work with many chemical medicines to boost their antitumor activity, as we mentioned before (30). Furthermore, DHA seems to have no impact on the chemotherapy sensitization of normal tissues or non-tumor tissues compared to tumors (31–34). Human endothelial cells, fibroblasts, and PBMC were neither sensitive to arsenic trioxide nor DHA, and the same was the combination of them. However, DHA exacerbated arsenic trioxide toxicity toward cancer cells. At the same time, studies declaimed that DHA lowered the sensitivity of normal cells that surrounded cancer cells. It was reported that the transformed fibroblasts became 19 times more sensitive to cytarabine after exposure to DHA, while the normal fibroblasts dropped to 1/4 of the original (35).

DHA was a non-toxic nutrient, the human consumption has been granted GRAS (generally recognized as safe) status by the FDA. *In vivo* experiments, DHA and IR combined sharpened the tumor volume and decreased the tumor cells in pathological tissues. Though there was not quantitative evidence showing that the DHA concentration level increased in the tumor tissue, we could affirm this effect according to the following reason: DHA was reported to be stable in human plasma total lipids when the intake of DHA was >1000 mg/d, whereas at <1000 mg/d, the DHA plasma dose-response relation was linear (23). The mean plasma half-time of DHA was 3.0 ± 0.2 d while supplemented with 1.4 g/d and therefore the plasma DHA concentration must have increased before the X-ray targeted radiotherapy (36). No statistical significance might appear because the gavage administration was not a natural feeding method. Applying the mice with food supplemented with PUFAs may improve the experiment.

Notwithstanding numerous studies pointing out that cancer cells have many molecular targets, the studies on DHA and PPAR-γ did not cohere (26, 37). The latest study upon a clinical trial conducted in Iran in 2014 indicated that an 8-weeks supplement of DHA would improve the *PPAR-*γ activity. Similarly, our results revealed that DHA remarkably aggravated *PPAR-*γ expression of irradiated esophagus cancer cells.

*PPAR-*γ is a widely distributed transcription factor. Several *in vivo* and *in vitro* studies have proved that *PPAR-*γ linked with a reduction of β-catenin levels, which plays a significant role in tumorigenesis (38–40). Thus, *PPAR-*γ seems to should be divided into the tumor suppressor genes family. DHA is a natural *PPAR-*γ activator, so it is accessible to understand why *PPAR-*γ is activated after the DHA supplementation esophagus cancer cell is irradiated. What's more, irradiation enhanced the expression of *PPAR-*γ after exposure to DHA. *PPAR-*γ is a crucial factor in adipogenesis. Ectopic expression of *PPAR-*γ could lead to adipose-specific gene expression and trigger the lipid droplet accumulation (41). Interestingly, irradiation was reported to result in the gathering of lipid droplets intracellularly in the prostate cancer cells (42). understandably, the expression of *PPAR-*γ after being treated with DHA and X-ray was more evident than irradiated only, although the mechanism remains unclear.

Our research illustrated that the radiation sensitization of DHA was partly attenuated by the *PPAR-*γ inhibitor, which means PPAR-γ may involve in the mechanism. More analyses are necessary to figure out the specific pathway. Besides this, we are very curious about the variation of autophagy and immunogenic death caused by DHA and radiation combination. Furthermore, the lipid oxidation, cell apoptosis and DNA damage level haven't been detected in the mice tumor cell xenograft experiment. We would absolutely incorporate them into our in-depth study scheme.

*PPAR-*γ activators have been used as a monotherapy in several advanced carcinomas, with no notable improvement observed (43). Our experiments indicated that DHA could improve the radiosensitivity of esophagus cancer cells and improve the expression of *PPAR-*γ. Deficiencies demonstrated in our research that whether DHA would affect the radiosensitivity of normal esophageal epithelial cells remains further study. Nonetheless, multimodality combination therapy is a tendency for co-therapy nowadays. Next, we will design an experiment about the clinical trial of DHA and radiation combination therapy for patients with esophageal squamous cancers.

## Conclusions

In conclusion, the present study focused on the potential combination therapy of irradiation and DHA. Moreover, a novel critical factor, *PPAR-*γ, was put forward to take the participant into this synergism. As DHA is a convenient and safe health care product, a new adjuvant treatment is on the agenda as long as our subsequent clinical trials suggest positively.

## Materials and Methods

### Reagents and Materials

We purchased DHA and LA powders from Sigma-Aldrich (St Louis, MO, USA). CCK-8 kit was bought from Beyotime (Shanghai, China). The stock solution of DHA and LA was prepared in the corresponding solvent described previously. These chemical ingredients were dissolved in the complete growth medium for working solutions. *PPAR-*γ antibody was ordered from Abcam (Cambridge, UK). Mifobate and GW9662 were purchased from Sigma-Aldrich (St Louis, MO, USA).

### Cell Line

The human esophagus cancer cell lines TE-1 and TE-10 were given by Professor Zhang at Suchow University as a gift. They were cultivated in RPMI Medium mixed with 10% heat-inactivated fetal bovine serum and 2% Penicillin-streptomycin.

### Cell Treatment With DHA/LA Together With Irradiation

TE-1 and TE-10 were treated with DHA/LA for 24 h before a single RT of 4Gy and the working concentrations were 150 and 100 mM.

### Cell Viability Assay

Cell viability was assessed using the CCK-8(cell counting kit 8) assay. Cells were plated into 96-well plates (6000/well), then treated with DHA/LA at 0, 25, 50, 100, and 200 μM for another 24 h on the second day. CCK-8 was diluted at 1:10 and then added to these plates (100 μL/plate). Microplate Reader was used to measure cell viability after a 30 min incubation.

### Clonogenic Assay

The cloning experiment was carried out in light of what was reported earlier. Simply put, TE-1 and TE-10 cells were plated into 6-well plates according to different cell numbers. Twenty four hours later, they were treated with DHA dissolved in the medium for 24 h before getting 0, 0.5, 1, 2, and 4Gy single doses of X-rays. After 14 days culturing, cells were fixed with 4% paraformaldehyde for 15 min then stained with crystal violet for 30 min. Clonings that formed after different treatment were caculated and normalized to the unirradiated cells.

### Cell Apoptosis Assay

Cells were pretreated with DHA and LA for 24 h before got irradiation. Another 24 h later, cells were collected and centrified at 800 rpm, 5 min to move the supernatant. Apoptosis was measured using the 7-AAD/Annexin-V double staining apoptosis kit (BD Biosciences, Franklin Lakes, NJ) by flow cytometry (BD Biosciences). The Annexin-V+/7-AAD- cells were in the early phase of the apoptotic process; the Annexin-V+/7-AAD+ cells indicated late apoptosis. The percentages of both groups of cells were computed. Each group was set up in triplicate.

### Cell Cycle Analysis

Both floating and attached cells were collected by centrifing at 800 rpm, 5 min after 24 h of irradiation. Cells were fixed with 70% ice-cold ethanol and then treated with 0.25 mg/ml RNase A and 50 μg/ml propidium iodide (PI) for 30 min at 37°C. For flow cytometry, 10,000 cells per sample were collected.

### Immunofluorescence Assay

The immunofluorescence staining technique was used to detect the DNA damage and lipid peroxidation after the irradiation. The fluorescent dyes used were as follows: DAPI, FITC, BODIPY. Cells were fixed with 4% paraformaldehyde for 15 min, then stained with different dyes at specified concentrations according to the instructions. Before observing under the microscope, cells were stained with DAPI dying that could illustrate the cell nucleus. Each group collected 10 photos containing at least ten cells per visual field. γ-H_2_AX foci number/cell count and comet tail length/ total length were counted to compare the differences.

### Western Blot Assay

Western blot was performed as standard schedule. The antibodies used in the experiment were *PPAR-*γ and *PPAR-*δ, and all the results were repeated three times. The relative grayscales of protein expression were normalized against the quantity of an internal control gene GAPDH (Santa Cruz Inc. California, USA).

### Mice Xenograft Experiment

Five-week-old Balb/C nude mice (male) were purchased from Si-Laike Experimental animal Company (Shanghai, China) and used in this video experiment. All animal experiments complied with the ARRIVE guidelines and were carried out following the National Institutes of Health guide for the care and use of laboratory animals (NIH Publications No. 8023, revised 1978). Every mouse was inoculated with TE-1 cells (10 × 10^5^) subcutaneously and they were randomly divided into six groups (*n* = 4). They received one of the following treatments while the tumors volume reached 100mm^3^: (1) intragastric administration of DHA(500mg/Kg·d); (2) intragastric administration of LA(500mg/Kg·d); (3) intragastric administration of ddH_2_O; (4) the combination of DHA and irradiation; (5) the combination of LA and irradiation; (6) the combination of ddH_2_O and irradiation. Tumors volume and mice weight were measured every 2 days right after treatment was given, and the Tumors got a single dose of 4Gy X-rays at day 7.

### Hematoxylin and Eosin Staining

The xenografts were fixed into 10% neutral-buffered formalin and paraffin-embedded. The tumor blocks were cut apart into 4 μm sections, then stained with an HE dyestuff kit (ZSGB-Bio, Beijing, China). The pathological sections were observed under Leica microscope. All nude mouse experiments were approved by the ethics committee of Soochow University.

### Statistical Analysis

All of the values we mentioned before were expressed as mean ± s.e.m. of three independent experiments. We used Graphpad Prism 8(Graphpad Software, San Diego, CA, USA) to calculate the unpaired two-tailed Student's *t*-test and one-way analysis of variance. Survival curves were assessed based on the Kaplan-Meier method and compared using a log-rank test. The difference among groups was regarded as meaningful when the *P*-value was <0.05.

## Data Availability Statement

The raw data supporting the conclusions of this article will be made available by the authors, without undue reservation.

## Ethics Statement

The animal study was reviewed and approved by Soochow University.

## Author Contributions

YJ: conceptualization, methodology, project administration, supervision, and writing-review & editing. YX: data curation. YY: formal analysis, investigation, and writing-original draft. LW: resources. CZ: software. AN: validation. LZ: visualization. All authors contributed to the article and approved the submitted version.

## Conflict of Interest

The authors declare that the research was conducted in the absence of any commercial or financial relationships that could be construed as a potential conflict of interest.

## Publisher's Note

All claims expressed in this article are solely those of the authors and do not necessarily represent those of their affiliated organizations, or those of the publisher, the editors and the reviewers. Any product that may be evaluated in this article, or claim that may be made by its manufacturer, is not guaranteed or endorsed by the publisher.

## Abbreviations

DHA: docosahexaenoic acid
LA: linoleic acid
ESCC: esophageal squamous cell carcinoma
EA: esophageal adenocarcinoma
RT: radiotherapy
PUFAs: polyunsaturated acid.
